# Anemia and Red Blood Cell Abnormalities in HIV-Infected and HIV-Exposed Breastfed Infants: A Secondary Analysis of the Kisumu Breastfeeding Study

**DOI:** 10.1371/journal.pone.0141599

**Published:** 2015-11-03

**Authors:** Collins Odhiambo, Clement Zeh, Pascale Ondoa, Paul Omolo, Benta Akoth, Humphrey Lwamba, Richard Lando, John Williamson, Juliana Otieno, Rose Masaba, Paul Weidle, Timothy Thomas, KiBS Study Team

**Affiliations:** 1 Centre for Global Health Research, Kenya Medical Research Institute, Kisumu, Kenya; 2 Division of HIV/AIDS Prevention, U.S. Centers for Disease Control and Prevention (CDC), Kisumu, Kenya; 3 Center for Poverty-related Communicable Diseases, Department of Internal Medicine, Center for Infection and Immunity, Amsterdam Institute for Global Health and Development, University of Amsterdam, Amsterdam, Netherlands; 4 Jaramogi Oginga Odinga Teaching and Referral Hospital, Kisumu, Kenya; 5 Family Health International, Nairobi, Kenya; 6 Division of HIV/AIDS Prevention, National Center for HIV, Viral Hepatitis, STD, and TB Prevention, CDC, Atlanta, GA, United States of America; University of Pittsburgh Center for Vaccine Research, UNITED STATES

## Abstract

**Background:**

Anemia results in increased morbidity and mortality, underscoring the need to better understand its pathophysiology amongst HIV-exposed and infected children in sub-Saharan Africa, the region where most infant HIV exposure and infections occur.

**Methods:**

This analysis used samples obtained from children in the Kisumu Breastfeeding Study (KiBS). KiBS was a longitudinal phase IIB, open-label, one-arm clinical trial, designed to investigate the safety, tolerability and effectiveness of a maternal triple-antiretroviral (ARV) regimen for prevention of mother-to-child transmission (PMTCT) of HIV, during late pregnancy and early infancy while breastfeeding. Blood samples from 482 children were obtained at birth, 2, 6, 10 and 14 weeks and 6, 9, 12, 18 and 24 months. Severity of anemia was graded using the NIH Division of AIDS (DAIDS) toxicity tables. We describe the proportion of children with anemia and anomalies in red blood cell parameters at various time points over 24 months and compare rates of anemia between HIV-infected and HIV-uninfected children and by mothers’ ARV regimen and infant malaria infection.

**Results:**

The proportion of children with anemia significantly increased after the breastfeeding period in both HIV-infected and HIV-uninfected children with higher proportion among HIV-infected children compared to HIV-uninfected children (RR: 1.72; CI: 1.22–2.44, p = 0.002). Maternal triple-antiretroviral regimen was not associated with infant anemia (p = 0.11). There was no significant difference in mean hemoglobin between HIV-uninfected children with and without malaria at each time point except at 24 months.

**Conclusion:**

A relatively lower proportion of children with severe anemia during the breastfeeding period suggest that exposure to mother’s triple antiretroviral combinations through breast milk, posed minimal risk of hematologic toxicity.

## Introduction

The World Health Organization (WHO) estimates that 3.4 million children younger than 15 years of age are infected with human immunodeficiency virus (HIV) worldwide, with the majority (92%) living in sub-Saharan Africa [1]. Most HIV infections in children occur via mother-to-child transmission (MTCT) and can be prevented by administration of antiretroviral (ARV) drugs during pregnancy and during the breastfeeding period in settings where breastfeeding is practiced among HIV-infected mothers [2–6]. Anemia in children is usually multifactorial; it can result from infectious diseases, nutritional deficiencies, and genetic factors, and the relative significance of each varies by geographic location, season and age [7, 8] and contributes to pediatric morbidity and mortality [9]. The pathophysiology of anemia in children is complex and can be difficult to interpret in the setting of the dynamic changes associated with normal hematological development [10]. In resource-poor and tropical settings, anemia is mainly caused by underlying nutritional deficiencies and endemic parasitic infections, such as malaria and helminthes, which lead to red blood cell destruction, decreased production or loss. In sub-Saharan Africa, hemoglobinopathies such as sickle cell disease represent an additional cause of anemia [11].

HIV infection in children is often complicated by anemia, which can result from the direct effect of HIV on bone marrow cells [12, 13], HIV-related opportunistic infections, certain ARVs, or HIV-unrelated conditions [7, 11, 12, 14]. The negative impact of HIV on anemic children worsens with the duration of HIV infection and the risk of anemia-associated morbidity and mortality is increased [10, 15]. In the context of HIV-associated anemia, zidovudine, given to children as treatment or for prevention of mother-to-child transmission (PMTCT) of HIV, can result in anemia through suppression of erythropoiesis [16, 17].

Anemia results in the decreased capacity of red blood cells to transport oxygen throughout the body and may be associated with general signs and symptoms including weakness, fatigue, lassitude, tachypnea and tachycardia [11]. Moderate anemia leads to a reduction in the quality of life; severe anemia can result in heart failure and death [18]. Whilst low hemoglobin levels represent the hallmark of anemia, other red cell indices such as mean corpuscular volume (MCV), red cell distribution width (RDW) and the red blood cell (RBC) count, in addition to other biochemical markers of iron status like serum ferritin and transferrin, provide further information on the pathophysiology and etiology of anemia. Based on the red blood cell shape and mode of reduction, anemia is classified either morphologically as normocytic, microcytic or macrocytic or pathophysiologically as excessive destruction, loss or diminished red cell production [11].

We evaluated episodes of anemia among HIV-infected and HIV-uninfected but exposed children whose mothers took triple-ARV prophylaxis during pregnancy (from 34–36 weeks gestation) and through 6 months of breastfeeding in a resource-limited setting where malaria, helminths and nutritional deficiency [16] are common causes of anemia. We investigated the effect of maternal triple-ARV prophylaxis and childhood malaria on red blood cell parameters.

## Materials and Methods

### Study population and drug regimen

The samples used in this analysis were obtained from children participating in the Kisumu Breastfeeding Study [6], a phase IIB, open-label, one-arm clinical trial that investigated the use of maternal triple-ARV prophylaxis for PMTCT. The study methods have been described previously [6]. In summary, HIV-1-infected pregnant women were enrolled at 34–36 weeks gestation and were counseled to exclusively breastfeed for 5.5 months then wean rapidly by 6 months post-partum. Screening evaluation included CD4+ T-lymphocyte count and complete blood count. Women with hemoglobin <7.0g/dL were excluded. Women were enrolled between July 2003 and November 2006. Follow up was completed in February 2009. The study intervention initially consisted of a maternal ARV regimen, zidovudine, lamivudine and nevirapine given from 34 to 36 weeks gestation through 6 months postpartum while breastfeeding regardless of CD4 count. Following the 2007 Food and Drug Administration advisory regarding increased hepatotoxicity among women with CD4 count ≥250 cells/mm^3^ initiating nevirapine-based regimen, the regimen was revised to use nelfinavir instead of nevirapine for women with a baseline CD4 count of ≥250 cells/mm^3^. Children received a single dose of nevirapine within 72 hours of birth. Mothers received cotrimoxazole throughout the study (except from 38 weeks gestation to delivery) while children received cotrimoxazole from 6 weeks until cessation of breastfeeding (at 6 months for most) and determined to be HIV-1-negative. Women meeting the WHO treatment initiation criteria (CD4<250 cells/mm^3^) at initiation or subsequently through the study period were continued on ARVs. Mother and infant pairs were followed for 24 months postpartum, and all mothers and HIV-1-infected children were managed and treated according to the national guidelines and linked to HIV care and treatment centers at exit from the study. Most HIV-1-infected children were initiated on triple-ARV combination therapy (containing zidovudine) based on the Kenya ministry of health guidelines.

### Blood sample collection and laboratory testing

Infant whole blood samples (5ml) were collected in EDTA vacutainer tubes (Becton Dickinson, San Jose, CA, USA) at each scheduled visit. This was used for complete blood count and to prepare dried blood spots for HIV-1 diagnosis routinely. Scheduled infant visits were at delivery (0–7 days), 2, 6, 10, 14 weeks, and 6, 9, 12, 18, and 24 months. Blood was not drawn from children with known hemoglobin less than 5g/dL unless this was done immediately prior to a scheduled transfusion.

Complete Blood count (CBC): was done using Coulter ACT 5diff CP analyzer (Beckman Coulter, France). Red blood cell indices included hemoglobin (Hgb), hematocrit (hct), mean corpuscular volume (MCV), mean corpuscular hemoglobin (MCH), red blood cell count and red blood cell distribution width (RDW) were determined for each infant at all study time points. The 1994 pediatric toxicity tables of the Division of AIDS (DAIDS), US National Institutes of Health was used to assess the degree of Hgb toxicity (graded 1–4 depending on degree of severity) [19]. Abnormal laboratory findings at each study time point were confirmed by repeat testing within 72 hours. All critical values (Grade 3&4) were reported within an hour to the study clinician.

HIV diagnosis: HIV-1 DNA PCR was performed on infant dried blood spots using the Roche Amplicor version 1.5 (Roche Diagnostic System, Branchburg, New Jersey, USA) as previously described [6]. The evaluation was done in real-time on all children’s specimens at 14 weeks, 6 and 9 months postpartum. For specimens obtained at 18 and 24 months postpartum, enzyme-linked immunosorbent assay (ELISA) was used instead with confirmation of positive results by HIV-1 DNA PCR on the Roche Amplicor version 1.5 (Roche Diagnostic System, Branchburg, New Jersey, USA). For specimens testing HIV positive at 18 and 24 months, we sequentially tested previously collected samples by PCR to determine the time of first HIV positivity [6].

Clinical Malaria diagnosis: Malaria infection was assessed whenever the infant was ill or anemic during scheduled and unscheduled study visits. Thick blood smears were prepared by adding 5μL of whole blood onto clean glass slides. After air drying, blood slides were stained using 10% Giemsa for 10 minutes. Using high power, 50 fields were examined by a trained microscopist before declaring a slide to be negative.

### Definition and clinical management of adverse events

Due to the physiological changes that occur in childhood that lead to variations in the level of hemoglobin, the criteria for defining anemia changes with age. DAIDS categorizes severity of anemia according to age into grade 1 (mild), grade 2 (moderate), grade 3 (severe) and grade 4 (life threatening) [19]. Hemoglobin results that were consistently grade 3 to 4 were reported as severe adverse events. Data from unscheduled study visits which involved a diagnosis of anemia were also included. Investigation for anemia at unscheduled visits was undertaken for a subset of children based on clinical assessment of the suspected adverse event. All cases of severe anemia were treated for underlying causes and/or transfused if hemoglobin was graded as severe or life threatening with signs of congestive heart failure. The response to treatment was evaluated at a follow-up visit within 2 weeks by measuring hemoglobin concentration in addition to clinical examination. Children were treated based on the Kenya ministry of health guidelines.

### Statistical analysis

All laboratory parameters were summarized by their means and standard deviations. Percentages were reported for categorical variables. The Mann-Whitney rank-sum test [20] was used to compare red blood cell parameters by visit for the following groups: HIV-infected versus HIV-uninfected, malaria-infected versus malaria-uninfected, maternal nevirapine-based versus nelfinavir-based triple- ARV prophylaxis, and HIV-1-infected children on treatment versus those not on treatment. An exact Pearson’s chi-square test was used to compare anemia proportions at a particular visit between HIV-1–infected children and HIV-1-uninfected children by visit. Pearson’s exact chi-square test was also used to compare the proportion of malaria case s during ARV intervention and post-intervention.

Log-binomial regression models [21] were used to fit time trends in malaria and anemia prevalence by incorporating covariates for HIV-1-infection group, infant age, and their interaction. Log-binomial regression was also used to model anemia proportions post-breastfeeding with the predictors HIV status, malaria status and their interaction. Linear regression was used to model hematological parameters post-breast feeding controlling for co-morbidity group. Generalized Estimating Equations (GEE) with an ‘independent’ working correlation matrix [22] was used in the above regression models to account for intra-individual correlation due to repeated observations on the same infant. Data were analyzed using SAS (SAS system for Windows 9.2; SAS, Inc., Cary, NC).

### Ethical approval

This study and the consenting procedures were approved by the Ethical Review Committee of the Kenyan Medical Research Institute as well as the Institutional Review Board of the U.S. Centers for Disease Control and Prevention, Atlanta, GA, USA. Adult (≥18 years) pregnant women provided written informed consent while pregnant minors (≥15 and <18 years) had to receive written guardian consent as well as provide their assent. Consent on behalf of the infants enrolled was written and signed by the mothers and/or guardian.

## Results

### Characteristics of the study population

Five hundred and two live children were born to 500 HIV-1-infected women in the study between July 2003 and January 2007; 54% were male. Twenty children withdrew or died before the first scheduled study visit at 0–7 days postpartum. Thus, 482 children with at least one laboratory result were included in this analysis, of which 284 (59%) and 198 (41%) were exposed to maternal nevirapine-based and nelfinavir-based ARV prophylaxis respectively, in utero and via breast feeding.

HIV infection: Overall, 32 children were diagnosed with HIV-1 infection over the 24-month follow-up period. A majority of HIV diagnoses occurred within 6 months of life (n = 24), three between 6 and 12 months, four between 12 and 18 months and one after 18 months. Seven HIV-1-infected children died, and two withdrew during the study period. Seven children met the criteria for treatment initiation during follow-up and were initiated on a zidovudine-based regimen.

Clinical malaria: The proportion of children with clinical malaria at each visit was less than 5% up to 6 months postpartum, after which it more than doubled and remained high through 24 months, when it was 16%. There was no difference in malaria episodes among children with HIV-1 compared with those without HIV-1 over the study period. Seven HIV-1-infected children (22%) had at least one episode of malaria during the study period, and one (3%) had more than one episode of malaria. In the HIV-1-uninfected, 125 (28%) children had at least one episode of malaria, with 36 children (7%) having multiple episodes during this period. HIV-1-infected children had malaria diagnosed at 8 (5%) out of a total of 158 visits where a smear was available during the 24 month follow-up period. In the HIV-1-uninfected children, malaria was diagnosed at 168 (5%) visits out of a total of 3275 visits where a smear was available. Episodes of malaria were significantly lower in children of mothers on a nelfinavir-based regimen 36/2137 visits (2%) compared to those whose mothers were on a nevirapine-based regimen, 140/1296 visits (11%; p <0.01).

### Frequency of anemia among children

The proportion of children with anemia increased over time in HIV-1-infected and HIV-1-uninfected children and was highest at 24 months postpartum (Table 1). The proportion of children with normal hemoglobin declined from over 70% at 6 months of age to less than 50% by 12 months of age in HIV-1-uninfected children. For HIV-1-infected children, the proportion of normal hemoglobin declined from over 90% at 6 weeks of age to less than 50% by 14 weeks of age (Table 1).

**Table 1:** Percent of HIV-uninfected and HIV-infected infants with normal and abnormal hemoglobin, Kisumu Breastfeeding study, Kisumu, Kenya, July 2003- February, 2009.

		Age of child (Months)							
	Grading	0-7d	2wks	6w	14w	6mo	12mo	18mo	24mo
HIV- uninfected infants	n	470	456	438	427	420	371	343	361
	Normal	87.9	71.5	89.2	75.1	71.5	48.5	46.8	31.2
	Grade 1	5.7	17.8	8.3	18.8	20.9	26.6	29.9	29.5
	Grade 2	3.6	10.5	2.5	6.1	6.9	23.0	22.1	37.6
	Grade 3&4	2.8	3.0	0	0	0.7	1.9	1.2	1.7
HIV- infected infants	n	12	18	19	21	23	22	21	20
	Normal	90.9	61.1	94.4	47.4	45.0	31.8	40.9	9.5
	Grade 1	0	5.5	5.5	36.8	35.0	31.8	9.1	38.1
	Grade 2	9.1	22.2	0	15.8	20.0	31.8	45.4	47.6
	Grade 3&4	0	11.1	0	0	0	4.5	4.5	4.8

Note: All infants identified with anemia received vitamins with iron supplementation, regardless of the grade of anemia, according to international guidelines.

#### Anemia in HIV-infected versus HIV-uninfected children

Both HIV-1-infected and HIV-1-uninfected children had CBC performed at each scheduled visit. Overall, the cumulative episodes (cases) of anemia at each time point among HIV-1-infected children (46 out of 219 visits (21%)) were significantly different from those among HIV-1-uninfected children (463 out of 3214 visits (14%)) (p = 0.01). In total, 49 children, accounting for 10% of total enrollment, had symptomatic severe anemia (grade 3 or 4) at some time point during the study follow-up period. However, the proportion of hospitalized cases of severe anemia was comparable between HIV-1-infected and HIV-1-uninfected children (3 children (9%) versus 46 children (9%), respectively). Cases of severe anemia peaked during the 6 to 12 month period (n = 19; 39%), coincident with cessation of breastfeeding and rise in malaria infections. There was an increased likelihood of progression to grade 2 or greater anemia in HIV-1-infected children compared to HIV-1-uninfected children after 6 months of age (Table 1) (RR: 1.72; CI: 1.22–2.44, p<0.01). Among the severe cases of anemia, four were transfused, and one died.

#### Evolution of red blood cell parameters over time in HIV-1-infected and HIV-1-uninfected children

**Fig 1:**
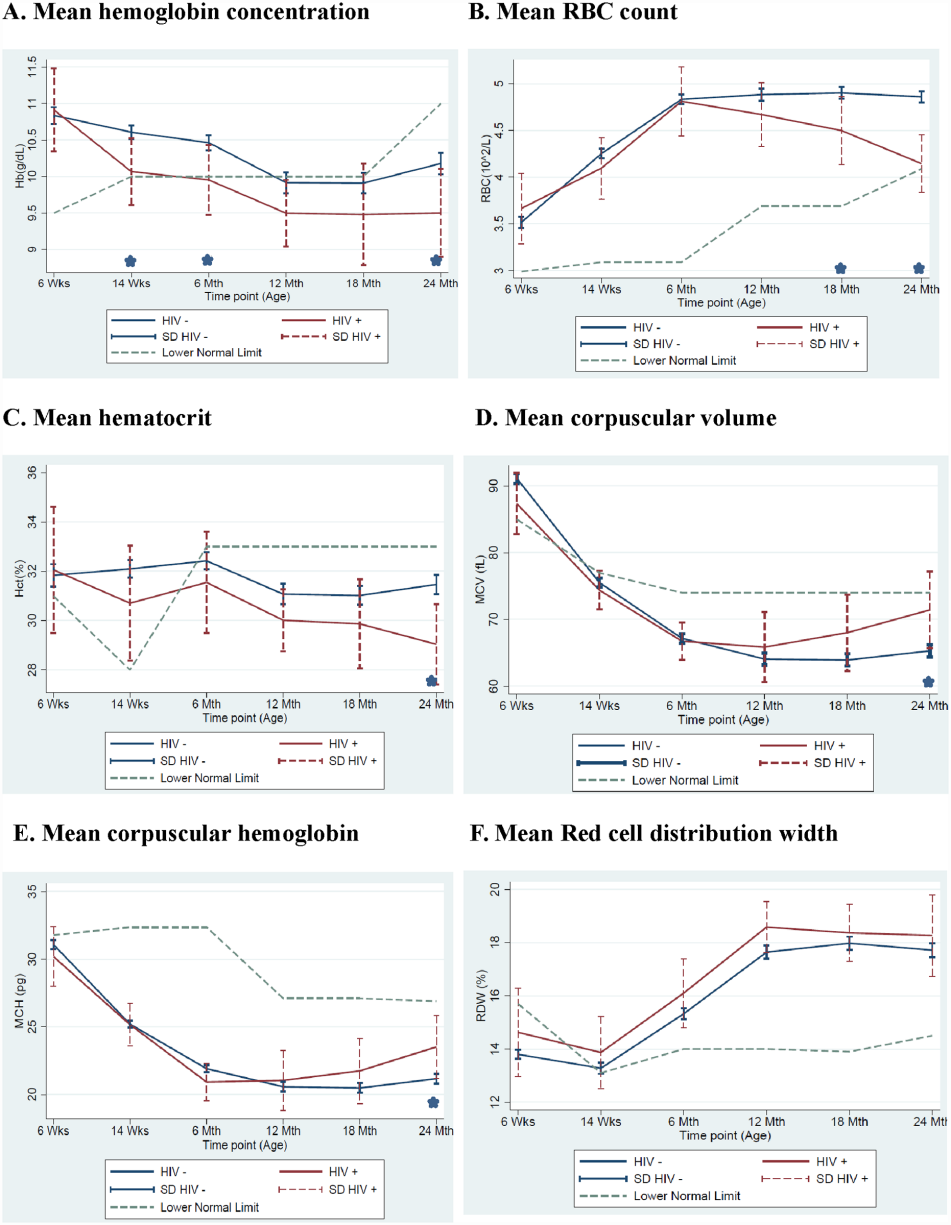
Comparison of mean A) hemoglobin concentration, B) RBC count, C) hematocrit, D) corpuscular volume, E) corpuscular hemoglobin and F) red cell distribution width by HIV-1 status in children, Kisumu Breastfeeding Study, Kisumu, Kenya, July 2003- February 2009. Dotted line represents the lower limit of normal for each parameter. (*p<0.05 between HIV-infected and HIV-uninfected children).

Mean Hemoglobin: Mean hemoglobin levels in the HIV-1-infected children were significantly lower than in uninfected children at 14 weeks (10.1 g/dL versus 10.6 g/dL; p = 0.025), 6 months (10.0 g/dL versus 10.52 g/dL; p = 0.031) and were lowest at 24 months of age (9.5 g/dL versus 10.2 g/dL; p = 0.03) (Fig 1A). In contrast to HIV-1-infected children, there was a non-significant increase in mean hemoglobin levels in the HIV-1-uninfected children from 18 months of age, but the level at 24 months remained below the lower limit of normal. Mean RBC and mean hematocrit (hct) followed a similar trend as hemoglobin with HIV-1-infected children having lower values than HIV-1-uninfected children. There was an increase in mean MCV and MCH over time in HIV-1-infected children and no difference in RDW between the two groups (Fig 1).

#### Association of malaria and HIV infection with infant red blood cell parameters

**Fig 2:**
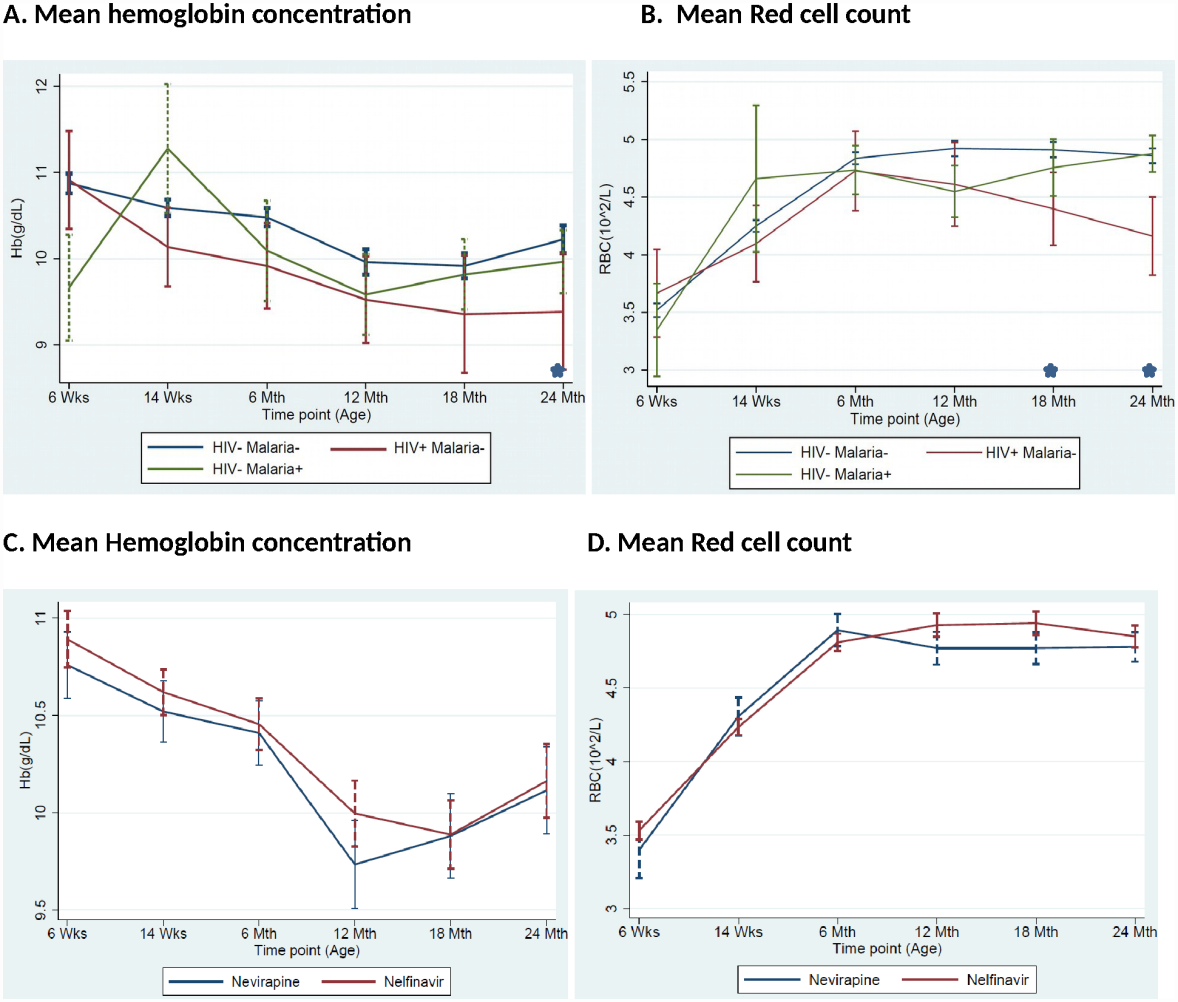
Comparison of mean A) hemoglobin concentration and B) RBC count by malaria infection status and mean C) hemoglobin concentration and D) RBC count by mother’s triple-antiretroviral prophylaxis regimen in children, Kisumu Breastfeeding Study, Kisumu, Kenya, July 2003- February 2009. (* p<0.05). HIV+ Malaria + omitted from graphs (2A and 2B) because of small sample size.

An infant was only considered malaria-infected at the particular visit with documented asexual stage parasitemia. Co-morbid status of the infants in this analysis is summarized in Table 2. HIV-1-uninfected children with malaria had lower mean hemoglobin concentration than HIV-1-uninfected children without malaria at each timepoint after 14 weeks of age (Fig 2A), though none were significantly different (p>0.05). A similar observation was made between HIV-1-infected children without malaria and HIV-1-uninfected children without malaria except at the 24 month time point (9.4g/dL versus 10.2g/dL; p = 0.02). The mean red blood cell counts for the HIV-1-uninfected children with malaria versus without malaria were not significantly different throughout the 24 months follow up (Fig 2B). The mean red blood cell counts of HIV-1-infected children without malaria were significantly lower than in HIV-1-uninfected children with malaria at 18 months (p≤0.01) and 24 months (p≤0.01) of age. The number of HIV-1-infected children with malaria was too small (n = 7) to allow for meaningful comparisons of hematologic indices by age with children in other categories of HIV/malaria status.

**Table 2:** HIV and Malaria co-morbidity among children, Kisumu Breastfeeding study, Kisumu, Kenya, July 2003- February, 2009.

Morbidity	0-7d	2wk	6wk	14wk	6mth	12mth	18mth	24mth
**HIV+/Malaria-**	12	18	19	20	22	20	20	17
**HIV-/Malaria+**	0	2	13	11	18	44	19	61
**HIV+/Malaria+**	0	0	0	1	1	2	1	3
**HIV-/Malaria-**	470	436	433	416	402	327	324	300

#### Association of triple-ARV therapy with infant red blood cell parameters

**Fig 3:**
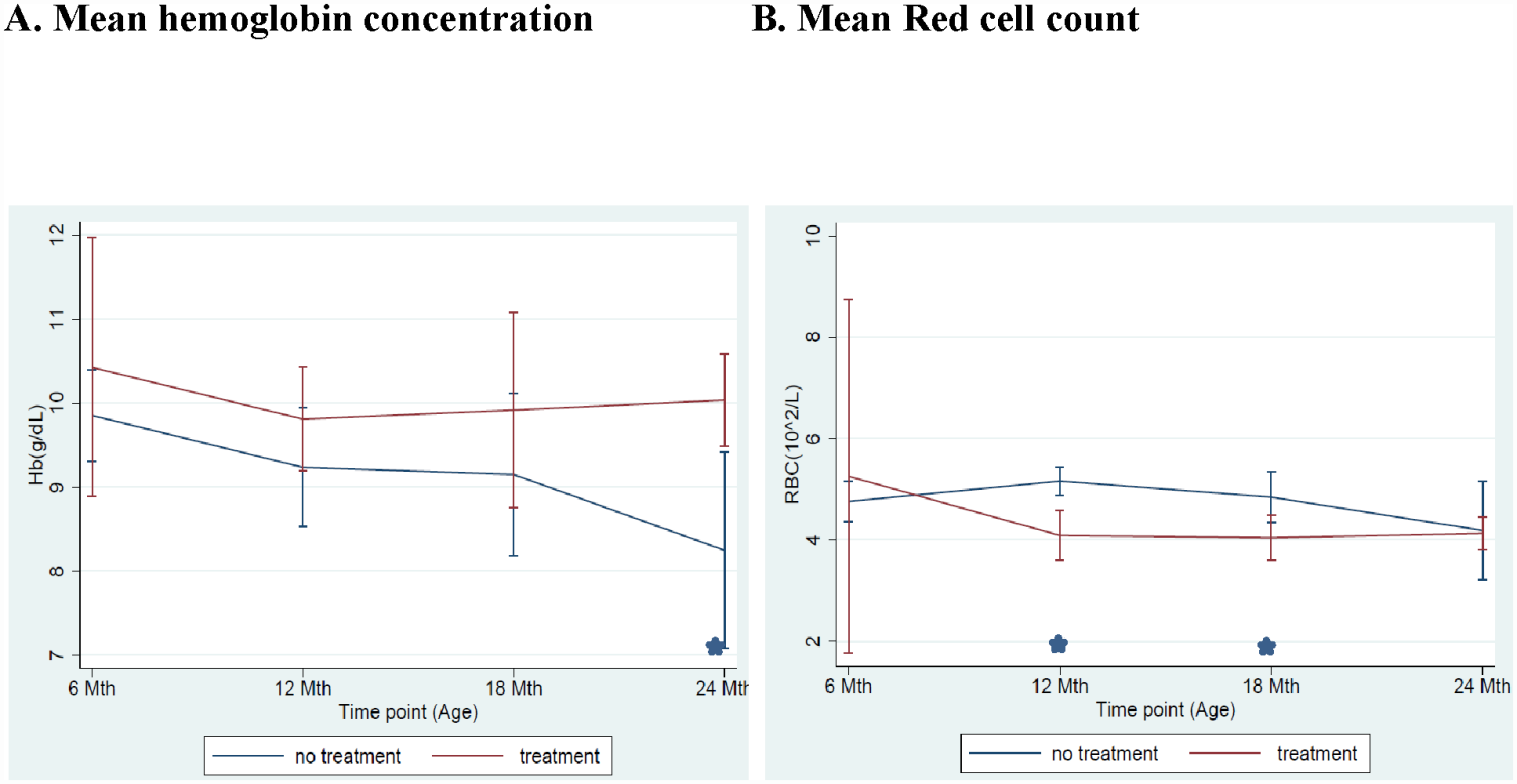
Comparison of mean A) hemoglobin concentration and B) RBC count by treatment status in HIV-1 infected children, Kisumu Breastfeeding Study, Kisumu, Kenya, July 2003- February 2009. (* p<0.05).

We evaluated the effect of either maternal nevirapine- or nelfinavir-based triple-ARV therapy on hematologic indices of the HIV—1uninfected infant parameters. The proportion of children with anemia over the study period was not associated with mothers’ triple-ARV therapy regimen (16% versus 14%; p = 0.11, for nevirapine versus nelfinavir-based regimen respectively). There was no difference in red blood cell indices in children whose mothers were on either of the two regimens (Fig 2A and 2B). Hemoglobin levels were lower and continued to decline in HIV-1-infected children not on treatment compared to those on triple-ARV combination therapy reaching significance by 24 months of age (8.3 g/dL versus 10.0 g/dL, p = 0.009) (Fig 3A). The red blood cell count in the treated children initially dropped, reaching a nadir by 12 months of age, after which the counts remained relatively stable through 24 months (Fig 3B). However, in the untreated children, red blood cell counts continued to decline after 12 months of age.

## Discussion

This analysis investigates the occurrence of anemia and hematological abnormalities among breastfed HIV-1-infected and HIV-1-uninfected children born to HIV-1-infected mothers in the Kisumu Breastfeeding study. Our results indicate that the proportion of children with anemia was higher in HIV-1-infected children than in HIV-1-uninfected children, consistent with previous findings in sub-Saharan Africa [7, 16], confirming the negative impact of HIV on hematologic parameters. While lower than the proportion in HIV-infected children, the increased proportion of anemia in HIV-1-uninfected children after the breastfeeding period suggests an effect of nutritional deficiency in such a resource-poor setting. Breastfeeding is associated with improved infant health and a four-fold protection against mortality [23, 24]. Early cessation of breastfeeding, is associated with increased infant mortality and morbidity [25]; however, in the context of HIV, the risk of HIV infection through breast milk necessitated earlier cessation (exclusive breastfeeding up to age 6 months) [26] than would be normally advocated. However, this study was conducted before option B+ (providing lifelong ART to all pregnant and breastfeeding women living with HIV) was considered by the WHO and helped inform those recommendations [27]. Other studies conducted in Italy and Zimbabwe confirm the negative ramifications of nutritional deficiency in infant HIV-associated anemia, as well as the difficulty of achieving normal hemoglobin value despite nutritional supplementation [7, 28]. The MCV declined through most of the study, potentially suggesting that iron deficiency may have been a factor in our study.

While malaria infected children had lower hemoglobin levels compared to malaria uninfected children, this was not statistically significant. The increase in malaria infections in HIV-uninfected children occurred primarily after 6 months of age, possibly as a result of waning maternally acquired antimalarial immunity [29, 30], and discontinuation of exposure to two drugs with known anti-malarial activity; cotrimoxazole (administered directly to children until 6 months and to mothers indefinitely) [31] and nelfinavir (administered to mothers who were breastfeeding up to 6 months) [32]. However, nelfinavir concentrations were minimal in the plasma of these breastfed children [33], and thus maternal nelfinavir is unlikely to have had an effect in this case. Hence, the difference could be attributed to cotrimoxazole withdrawal.

The present analysis allowed us to investigate the impact of ARVs on the occurrence of anemia during the PMTCT intervention. Zidovudine is known to have a toxic effect on hematopoiesis [17, 34] and neutrophil counts [35]. Our analysis included children not exposed to zidovudine (HIV-negative children from 6–24 months), allowing the assessment of the relationship between HIV infection and hematological parameters studied. The HIV-infected mothers were all given the same nucleoside reverse transcriptase inhibitors drugs; zidovudine and lamivudine with the additional drug being a protease inhibitor (nelfinavir) or a non-nucleoside reverse transcriptase inhibitor (nevirapine). Children were exposed to mothers’ triple-ARV therapy through breast milk until 6 months of age, after which breastfeeding ceased. Our observations do not support a significant role for maternal zidovudine in the development of anemia in HIV-exposed children. First, only HIV-infected children had significantly lower levels of hemoglobin concentration from 14 weeks through 6 months of age, during which all infants, regardless of HIV status, were exposed to zidovudine via breast milk. Secondly, MCV showed a trend towards microcytosis in both HIV-1-infected and uninfected infants suggesting that zidovudine was not present in sufficient quantities to induce macrocytosis, as might have been expected [13]. These data are in line with previous findings from the same population indicating that zidovudine concentration in breast milk and infant plasma was below biologically significant quantities [33]. Thirdly, most anemia cases occurred after cessation of breastfeeding, when there was a potential of ARV exposure, with peak number of cases between 6–12 months of age. Moreover, we observed a higher hemoglobin concentration in HIV-1-infected children placed on a zidovudine-based regimen (n = 11) compared to HIV-1-infected children not on treatment (n = 8). These children were not started on ARV treatment immediately after HIV was diagnosed based on the guidelines in force at that time in which ARV initiation was based on CD4 staging [36] Our finding of higher hemoglobin levels in these children is supported by studies in resource limited settings that demonstrate increased level of hemoglobin in adult patients on ART including those on zidovudine-based regimens [37–39]. We observed a lower occurrence of severe anemia during the first six months of age compared with a similar study in Botswana that implicated mothers’ triple-ARV therapy as a cause of increased infant anemia cases compared to a single-ARV regimen [40]. While the Botswana study provided one month of zidovudine prophylaxis to the newborn children, our study administered single-dose nevirapine at birth for PMTCT. It is possible that the increased occurrence of severe anemia in the Botswana study may have been due to infant zidovudine prophylaxis rather than maternal triple ARV combination therapy. Collectively, these data suggest that most anemia cases in our study were not caused by infant exposure to maternal zidovudine either in-utero or through breast milk. Our analysis also shows that red cell indices were not affected by either nevirapine or nelfinavir perinatal exposure or via breastfeeding as indicated by similar hematological indices at birth and throughout the follow up period for the two regimens. The hemoglobin profile was similar for both treatment groups and was comparable to previous observations in a study of HIV-uninfected children exposed to various combinations of ARV drugs [2]. These observations suggest that the use of triple-ARV therapy for PMTCT including breastfeeding does not significantly contribute to the development of infant hematologic abnormalities.

Most children with low hemoglobin levels had no corresponding clinical symptoms reported. This observation may have been due to misclassification associated with the use of the DAIDS toxicity tables which are derived from Western reference values known to differ from African values [41, 42]. Previous studies comparing normal reference values for hematologic parameters among African and US or European children indicate that there are differences between these groups in terms of normal values expected in healthy populations. Thus, there is need to establish locally-derived reference values for children that can be used for anemia management in western Kenya.

This analysis has several limitations. Firstly, there is no direct comparison group in this one-arm clinical trial. Secondly, we did not obtain a blood smear for malaria screening for all participants at all study visits; hence we may have underestimated the role of malaria in the observed anemia. Another limitation of this analysis was the low number of HIV-infected children. Although we did not assess the contribution of hemoglobinopathies, the prevalence of these genetic defects such as sickle cell disease (0.8%) is low in Kenya [43]. A major limitation was our inability to determine the specific etiology of anemia since we did not assess for levels of iron, iron binding capacity and ferritin among others.

In summary, anemia was most common among HIV-1-infected children, but occurred post breastfeeding, suggesting little contribution from the maternal combination ARV regimen. HIV-1-infected children on treatment also had better outcomes than HIV-1-infected children not on treatment, as indicated by significantly higher hemoglobin levels. Thus, prompt ARV treatment of HIV-infected children upon diagnosis may be associated with better hematological outcomes pending relevant evaluations of this result based on our longitudinal data analysis.

## References

[1] Joint United Nations Programme on HIV/AIDS (UNAIDS). Global Report: Report on the Global HIV/AIDS epidemic. UNAIDS 2010. Available: http://www.who.int/hiv/pub/global_report2010/en/. Accessed 2015 Sept 21.

[2] Feiterna-Sperling C, Weizsaecker K, Buhrer C, Casteleyn S, Loui A, Schmitz T, et al Hematologic effects of maternal antiretroviral therapy and transmission prophylaxis in HIV-1-exposed uninfected newborn infants. J Acquir Immune Defic Syndr. 2007; 45(1):43–51. 17356471 10.1097/QAI.0b013e318042d5e3

[3] Moodley D, Moodley J, Coovadia H, Gray G, McIntyre J, Hofmyer J, et al A multicenter randomized controlled trial of nevirapine versus a combination of zidovudine and lamivudine to reduce intrapartum and early postpartum mother-to-child transmission of human immunodeficiency virus type 1. J Infect Dis. 2003; 187(5):725–35. 12599045 10.1086/367898

[4] Nommsen-Rivers L, Heinig MJ. HIV transmission via breastfeeding: reflections on the issues. J Hum Lact. 1997; 13(3):179–81. 9341406 10.1177/089033449701300301

[5] Shaffer N, Chuachoowong R, Mock PA, Bhadrakom C, Siriwasin W, Young NL, et al Short-course zidovudine for perinatal HIV-1 transmission in Bangkok, Thailand: a randomised controlled trial. Bangkok Collaborative Perinatal HIV Transmission Study Group. Lancet. 1999; 353(9155):773–80. 10459957 10.1016/s0140-6736(98)10411-7

[6] Thomas T, Masaba R, Borkowf CB, Ndivo R, Zeh C, Misore A, et al Triple-antiretroviral prophylaxis to prevent mother-to-child HIV transmission through breastfeeding—the Kisumu Breastfeeding Study, Kenya: a clinical trial. PLoS Med. 2011; 8(3):e1001015 10.1371/journal.pmed.1001015 21468300 PMC3066129

[7] Miller M, Humphrey JH, Iliff PJ, Malaba LC, Mbuya NV, Stoltzfus RJ. Neonatal erythropoiesis and subsequent anemia in HIV-positive and HIV-negative Zimbabwean babies during the first year of life: a longitudinal study. BMC Infect Dis. 2006;6(1). 10.1186/1471-2334-6-1 PMC1361802 16390553

[8] Miller M, Stoltzfus RJ, Iliff PJ, Malaba LC, Mbuya NV, Humphrey JH. Effect of maternal and neonatal vitamin A supplementation and other postnatal factors on anemia in Zimbabwean infants: a prospective, randomized study. Am J Clin Nutr. 2006; 84(1):212–22. 16825698 10.1093/ajcn/84.1.212

[9] English M, Ahmed M, Ngando C, Berkley J, Ross A. Blood transfusion for severe anaemia in children in a Kenyan hospital. Lancet. 2002; 359(9305):494–5. 11853798 10.1016/S0140-6736(02)07666-3

[10] Calis J, van Hensbroek MB, de Haan RJ, Moons P, Brabin BJ, Bates I. HIV-associated anemia in children: a systematic review from a global perspective. AIDS. 2008; 22(10):1099–112. 18525255 10.1097/QAD.0b013e3282fa759f

[11] Calis J, Phiri KS, Faragher EB, Brabin BJ, Bates I, Cuevas LE, et al Severe anemia in Malawian children. N Engl J Med. 2008; 358(9):888–99. 10.1056/NEJMoa072727 18305266

[12] Bain B. Pathogenesis and pathophysiology of anemia in HIV infection. Curr Opin Hematol. 1999; 6(2):89–93. 10088638 10.1097/00062752-199903000-00006

[13] Moses A, Nelson J, Bagby GC Jr. The influence of human immunodeficiency virus-1 on hematopoiesis. Blood. 1998; 91(5):1479–95. 9473211

[14] Mueller B. Hematological problems and their management in children with HIV infection In: Pizzo PA, Wilfert CM, editors. Pediatric AIDS, the challenge of HIV infection in infants, children, and adolescents. Baltimore; Williams & Wilkins,1994 p. 591–601.

[15] van Eijk A, Ayisi JG, ter Kuile FO, Misore AO, Otieno JA, Kolczak MS, et al Malaria and human immunodeficiency virus infection as risk factors for anemia in infants in Kisumu, western Kenya. Am J Trop Med Hyg. 2002; 67(1):44–53. 12363063 10.4269/ajtmh.2002.67.44

[16] Semba R, Broadhead R, Taha TE, Totin D, Ricks MO, Kumwenda N. Erythropoietin response to anemia among human immunodeficiency virus-infected infants in Malawi. Haematologica. 2001; 86 (11):1221–2. 11694410

[17] Taha T, Kumwenda N, Gibbons A, Hoover D, Lema V, Fiscus S, et al Effect of HIV-1 antiretroviral prophylaxis on hepatic and hematological parameters of African infants. AIDS. 2002; 16 (6):851–8. 11919486 10.1097/00002030-200204120-00004

[18] Semba R, Martin BK, Kempen JH, Thorne JE, Wu AW, Ocular Complications of AIDS Research Group. The impact of anemia on energy and physical functioning in individuals with AIDS. Arch Intern Med. 2005; 165:2229–36. 16246988 10.1001/archinte.165.19.2229

[19] National Institutes of Health. Division of AIDS Table for Grading the Severity of Pediatric Adverse Events 1994 [cited 1 October 2010]. Available: http://rsc.tech-res.com/safetyandpharmacovigilance/gradingtables.aspx. Accessed 2015 Sept 21.

[20] Wilcoxon F. Individual comparisons by ranking methods. Biometrics Bulletin. 1945; 1(6):80–3.

[21] Wacholder S. Binomial regression in GLIM: estimating risk ratios and risk differences. Am J Epidemiol. 1986; 123:174–84. 3509965 10.1093/oxfordjournals.aje.a114212

[22] Hardin J. Generalized Estimating Equations. London: Chapman and Hall/CRC; 2003. ISBN 978-1-58488-307-4.

[23] Heinig M. Breastfeeding decisions. Pediatrics. 2002; 110(5):1033–4. 12415055 10.1542/peds.110.5.1033

[24] United Nations Children’s Fund. Unite for children, unite against AIDS campaign. UNICEF 2005 Available: http://www.unicef.org/aids/index_29444.html. Accessed 2015 Sept 21.

[25] Heinig M. The American Academy of Pediatrics recommendations on breastfeeding and the use of human milk. J Hum Lact. 1998; 14 (1):2–3. 9543949 10.1177/089033449801400102

[26] World Health Organization. Technical consultation on behalf of the UNFPA/UNICEF/WHO/UNAIDS Interagency Task Team on Mother–to-child Transmission of HIV. New data on the prevention of mother to child transmission of HIV and their policy implications Conclusions and recommendations. Geneva, Switzerland: World Health Organization; 10 11–23, 2000 Available: http://apps.who.int/iris/bitstream/10665/66851/1/WHO_RHR_01.28.pdf. Accessed 2015 Sept 21.

[27] World Health Organization. Programmatic Update. Use of antiretroviral drugs for treating pregnant women and preventing HIV infection in infants. 2012. Available: http://www.who.int/hiv/pub/mtct/programmatic_update2012/en/. Accessed 2015 Sept 21.

[28] Galli L, de Martino M, Rossi ME, Panza B, Farina S, Vierucci A. Hemochrome parameters during the first two years of life in children with perinatal HIV-1 infection. Pediatr AIDS HIV Infect. 1995; 6(6):340–5. 11361457

[29] Amaratunga C, Lopera-Mesa TM, Brittain NJ, Cholera R, Arie T, Fujioka H, et al A role for fetal hemoglobin and maternal immune IgG in infant resistance to Plasmodium falciparum malaria. PLoS One 2011; 6(4):e14798 10.1371/journal.pone.0014798 21532754 PMC3075246

[30] Riley E, Wagner GE, Akanmori BD, Koram KA. Do maternally acquired antibodies protect infants from malaria infection? Parasite Immunol 2001; 23(2):51–9. 11240896 10.1046/j.1365-3024.2001.00364.x

[31] Mermin J, E J, Liechty CA, Were W, Downing R, Ransom R, et al Effect of co-trimoxazole prophylaxis, antiretroviral therapy, and insecticide-treated bednets on the frequency of malaria in HIV-1-infected adults in Uganda: a prospective cohort study. Lancet. 2006; 367(9518):1256–61. 16631881 10.1016/S0140-6736(06)68541-3

[32] Mishra L, Bhattacharya A, Sharma M, Bhasin VK. HIV protease inhibitors, indinavir or nelfinavir, augment antimalarial action of artemisinin in vitro. Am J Trop Med Hyg. 2010; 82(1):148–50. 10.4269/ajtmh.2010.09-0427 20065012 PMC2803526

[33] Mirochnick M, Thomas T, Capparelli E, Zeh C, Holland D, Masaba R, et al Antiretroviral concentrations in breast-feeding infants of mothers receiving highly active antiretroviral therapy. Antimicrob Agents Chemother. 2009; 53(3):1170–6. 10.1128/AAC.01117-08 19114673 PMC2650559

[34] El Beitune P, Duarte G. Antiretroviral agents during pregnancy: consequences on hematologic parameters in HIV-exposed, uninfected newborn infant. Eur J Obstet Gynecol Reprod Biol. 2006; 128(1–2):59–63. 16876310 10.1016/j.ejogrb.2006.01.013

[35] Pacheco S, McIntosh K, Lu M, Mofenson LM, Diaz C, Foca M, et al Effect of perinatal antiretroviral drug exposure on hematologic values in HIV-uninfected children: An analysis of the women and infants transmission study. J Infect Dis. 2006; 194(8):1089–97. 16991083 10.1086/507645

[36] World Health Organization. Scaling up antiretroviral therapy in resource-limited settings: Treatment guidelines for a public health approach. 2003. Available: http://www.who.int/3by5/publications/documents/arv_guidelines/en/. Accessed 2015 Sept 21.

[37] Hoffmann CJ, Fielding KL, Charalambous S, Sulkowski MS, Innes C, Thio CL, et al Antiretroviral therapy using zidovudine, lamivudine, and efavirenz in South Africa: tolerability and clinical events. AIDS. 2008; 22(1):67 18090393 10.1097/QAD.0b013e3282f2306e PMC3724474

[38] Johannessen A, Naman E, Gundersen SG, Bruun JN. Antiretroviral treatment reverses HIV-associated anemia in rural Tanzania. BMC infect dis. 2011; 11(1):190. 21745396 10.1186/1471-2334-11-190 PMC3145581

[39] Kiragga AN, Castelnuovo B, Nakanjako D, Manabe YC. Baseline severe anaemia should not preclude use of zidovudine in antiretroviral-eligible patients in resource-limited settings. J Int AIDS Soc. 2010; 13 (1):42. 21047391 10.1186/1758-2652-13-42 PMC2991285

[40] Dryden-Peterson S, Shapiro RL, Hughes MD, Powis K, Ogwu A, Moffat C, et al Increased risk of severe infant anemia after exposure to maternal HAART, Botswana. J Acquir Immune Defic Syndr. 2011; 56(5):428–36. 21266910 10.1097/QAI.0b013e31820bd2b6 PMC3112252

[41] Buchanan A, Muro FJ, Gratz J, Crump JA, Musyoka AM, Sichangi MW, et al Establishment of haematological and immunological reference values for healthy Tanzanian children in Kilimanjaro region. Trop Med Int Health. 2010;15 (9):1011–21. 10.1111/j.1365-3156.2010.02585.x 20636301 PMC3024440

[42] Lubega I, Fowler MG, Musoke PM, Elbireer A, Bagenda D, Kafulafula G, et al Considerations in using US-based laboratory toxicity tables to evaluate laboratory toxicities among healthy malawian and Ugandan infants. J Acquir Immune Defic Syndr. 2010; 55(1):58–64. 20588184 10.1097/QAI.0b013e3181db059d PMC3033212

[43] Komba AN, Makani J, Sadarangani M, jala-Agbo T, Berkley JA, Newton CR, et al Malaria as a cause of morbidity and mortality in children with homozygous sickle cell disease on the coast of Kenya. Clin Infect Dis. 2009; 49 (2):216–22. 10.1086/599834 19514855 PMC2727464

